# St. George's Hospital and King Edward's Fund

**Published:** 1912-06-22

**Authors:** A. William West

**Affiliations:** Treasurer and Chairman, St. George's Hospital.


SIR,—The Hon. Secretaries of the King's Fund in their letter in yesterday's issue beg the whole question, and they have so far furnished no answer to my letters of May 13 and 22. The King's Fund has asserted, among other things, that St. George's Hospital spends 2½ to 2¾ times above the average of other hospitals upon certain work. St. George's Hospital maintain that this assertion is not true if all the facts are taken into consideration. The King's Fund has made the accusation and have been asked to produce the evidence on which it is based, but have not seen fit to do so; if they will do so before an independent committee St. George's Hospital is both willing and anxious to lay its evidence before the same committee.—Yours truly,

A. WILLIAM WEST, Treasurer and Chairman,

June 11. St. George's Hospital.

